# JTE-522, a selective COX-2 inhibitor, inhibits growth of pulmonary metastases of colorectal cancer in rats

**DOI:** 10.1186/1471-2407-5-26

**Published:** 2005-03-03

**Authors:** Hirotoshi Kobayashi, Hiroyuki Uetake, Tetsuro Higuchi, Masayuki Enomoto, Kenichi Sugihara

**Affiliations:** 1 Department of Surgical Oncology, Graduate School, Tokyo Medical and Dental University, 1-5-45 Yushima, Bunkyo-ku, Tokyo 113-8519, Japan

## Abstract

**Background:**

Epidemiological studies have shown that individuals who regularly consume NSAIDs have lower rates of mortality associated with colorectal cancer. Because COX-2 inhibitors prevent tumor growth through some mechanisms, we assessed the effect of JTE-522, a selective COX-2 inhibitor, on pulmonary metastases of colon cancer in a rat model.

**Methods:**

A suspension of 5 × 10^6 ^RCN-9 (rat colon cancer cells) was injected into the tail vein of 24 anesthetized male F344/DuCrj rats. Oral JTE-522 (0, 3, 10, or 30 mg/kg/day) was administered from the day before RCN-9 injection until the end of the study. Twenty-four days later, the lungs were removed from sacrificed rats and weighed. Pulmonary metastatic tumors were microscopically evaluated in the largest cross sections. We also performed immunohistochemical staining for both COX-2 and VEGF.

**Results:**

JTE-522 dose-dependently decreased lung weight (p = 0.001) and the size of pulmonary metastatic tumors (p = 0.0002). However, the differences in the number of metastatic tumors among 4 groups were insignificant. Significant adverse effects of JTE-522 were undetectable. Immunohistochemical staining showed high levels of both COX-2 and VEGF in pulmonary metastatic tumors.

**Conclusion:**

JTE-522 dose-dependently decreased the size, but not the number of pulmonary metastases. COX-2 inhibitors might block metastatic tumor growth, but not actual metastasis. Selective COX-2 inhibitors might be useful as therapeutic agents that inhibit the growth of metastatic tumors, as well as the tumorigenesis of colorectal cancer.

## Background

Colorectal cancer is a widespread fatal malignancy in the West [1] and its incidence in Japan is increasing [2]. Epidemiological studies have shown that regular doses of nonsteroidal anti-inflammatory drugs (NSAIDs) are associated with lower rates of colorectal polyps, colorectal cancer, and colorectal cancer-associated mortality. Waddell and Loughry initially reported that sulindac decreased the number of polyps in patients with familial adenomatous polyposis (FAP) [3]. The largest study of aspirin and colon cancer was undertaken by Thun *et al*. in 1991 [4]. They found that colon cancer mortality decreased among those who used aspirin, and that the protective effect was increased by increasing doses. NSAIDs inhibit prostaglandin production mainly by inhibiting cyclooxygenase (COX) that is expressed as COX-1 and COX-2 isoforms. Cox is a key enzyme, which converts arachidonic acid to prostaglandins. COX-1 is constitutively expressed in most tissues and is thought to be responsible for homeostatic functions. On the other hand, COX-2 expression is induced by various factors including cytokines, growth factors, and tumor promoters [5,6]. Many studies have indicated that COX-2 expression is associated with both the carcinogenesis and growth of colon cancer. COX-2 is overexpressed in 85% of human colorectal cancers [7]. Oshima *et al*. disclosed that COX-2 gene knockouts and a COX-2 inhibitor decreased the number of polyps of apcΔ716 knockout mice [8]. In addition, several studies have shown that the selective inhibition of COX-2 reduces colorectal tumorigenesis in various models of carcinogenesis. Reddy *et al*. found that the COX-2 inhibitor, SC-58635 (celecoxib), significantly suppressed colonic aberrant crypt foci and crypt multiplicity in rats [9]. This supports the hypothesis that COX-2 inhibitors are chemopreventive. Kawamori *et al*. showed that celecoxib inhibited both the incidence and multiplicity of colon tumors in a rat model [10], and another study showed that COX-2 expression in colon cancer cells increased metastatic potential [11]. We reported that increased COX-2 levels in human colorectal carcinomas correlated with both the size and invasiveness of tumors [12]. Tomozawa *et al*. demonstrated a correlation between COX-2 overexpression and the recurrence of colorectal cancer [13].

It is well known that colorectal metastasis occurs primarily in the liver and secondarily in the lung [14]. A laparotomy is necessary for making experimental model of liver metastasis in rats, but it is also a stressful event. On the other hand, it is less invasive to make lung metastasis by injection of tumor cells into the tail vein of rats. Thus, we considered that the experimental model of pulmonary metastasis was appropriate for the evaluation of the real effect of JTE-522, a selective COX-2 inhibitor, on hematogenous metastasis of colon cancer in rats. The aim of this study was to examine the effect of JTE-522 on hematogenous metastasis of colon cancer in a rat model.

## Methods

### Preparation of JTE-522

The selective COX-2 inhibitor, JTE-522 [15], was suspended in 0.5% carboxymethyl cellulose sodium salt (Wako Life Science Reagents, Osaka, Japan).

### Animals

Twenty-four 4-week-old male F344/DuCrj rats, weighing 50 to 70 g purchased from SLC (Shizuoka, Japan) were acclimatized for one week before use in this study. The rats were maintained in an environment with a controlled temperature (22 to 24°C), humidity (40 to 50%), and lighting (12 hours light-dark cycle) with free access to tap water and standard rodent chow (CE-2, Nihon Clea, Tokyo, Japan).

### Tumors

The RIKEN CELL BANK donated the rat colon cancer cell line, RCN-9, which was established by Inoue *et al*. after inbred F344/DuCrj rats were subcutaneously injected with neutralized 1, 2- dimethylhydrazine (DMH) [16]. The tumor cells were stored at -80°C. After thawing, tumor cells were washed in phosphate-buffered saline (PBS) and resuspended in RPMI-1640 (Sigma Chemical Co., St. Louis, MO) medium containing 10% heat-inactivated fetal bovine serum (FBS; Sigma) and 0.05% penicillin-streptomycin (Sigma). After repeated subculture, cell viability was assessed by Trypan blue dye exclusion and the cells were counted using a hemocytometer.

### Induction of lung metastases

F344 male rats were anesthetized with ether and then a tumor suspension containing 5 × 10^6 ^RCN-9 cells in 0.4 ml of PBS was injected into the tail vein.

### Pulmonary metastases of colon cancer in rats

Twenty-four rats were divided into four groups. Group 1 (control) was not given JTE-522. The other three groups all orally received daily doses of 3, 10 and 30 mg/kg JTE-522 (groups 2, 3 and 4, respectively) by gavage from the day before RCN-9 injection until the end of the study. The rats were sacrificed 24 days after injection and the lungs were removed and weighed. After 3-day formalin fixation, lungs were embedded in paraffin. Paraffin-embedded sections were cut into 3-μm serial sections, which stained with hematoxylin and eosin to evaluate the pharmacological effects of JTE-522 on pulmonary metastases. The number and the largest diameter of metastatic tumors were examined microscopically in the largest sagittal cross section. The largest metastatic tumor was selected after measuring the diameter of 10 tumors in each slide.

### Immunohistochemistry

Slides were immunohistochemically examined using the Universal Immuno-enzyme Polymer (UIP). Sections of 3 μm thick were cut from formalin-fixed and paraffin-embedded blocks of rat tissues. The slides were deparaffinized in xylene, rehydrated through a graded series of ethanols and washed with distilled deionized water. Antigen was retrieved by incubating the sections in 10 mmol/L citrate buffer (pH 6.0) in an autoclave (120°C) for 5 minutes. Thereafter, slides were cooled to room temperature and then washed with distilled deionized water. To quench endogenous peroxidase activity, slides were immersed in 3% H_2_O_2 _in methanol for 20 minutes. After a short rinse in phosphate-buffered saline (PBS), non-specific binding was blocked by incubating the sections in 10% normal goat serum for 10 minutes at room temperature. Thereafter, the slides were incubated with primary antibodies directed against COX-2 (rabbit polyclonal, Cayman Chemical, Ann Arbor, MI; dilution 1:250) for 2 hours or vascular endothelial growth factor (VEGF) (rabbit polyclonal, VEGF A-20: sc-152; Santa Cruz Biotechnology, Santa Cruz, CA; dilution 1:200) for 1 hour at room temperature. The slides were washed in PBS (3 × 5 minutes) and incubated with secondary antibody (N-Histofine^® ^Simple Stain MAX PO, Nichirei, Tokyo, Japan) for 30 minutes at room temperature. After washing in PBS, color was developed using 3, 3'-diaminobenzidine tetrahydrochloride (Sigma) in 50 mmol Tris-HCl (pH 7.5) for 5 minutes and counterstaining with 1% Mayer's hematoxylin. Sections known to show intense immunostaining for COX-2 and VEGF were included in each experiment, having been incubated with either primary antibody or PBS as positive and negative controls, respectively. The positive controls were always obviously stained and negative controls were never stained.

### Evaluation of immunostaining

The specimens immunostained for COX-2 and VEGF were independently scored by two blinded investigators. Both the distribution (the percentage of positive cells) and the intensity of staining were assessed in a semiquantitative fashion. Sections were scored, according to the method of Yukawa *et al *[17]. Both the number of positive cells and the intensity of staining were evaluated as follows. The number of positive cells: none, 0; focal (one third of cells stained), 1; multifocal (two third of cells stained), 2; and diffuse (most cells stained), 3. The intensity of staining: none, 0; mild (between 0 and 2), 1; and strong (clearly identified by × 40 magnification), 2. The score for distribution and intensity were added and graded as follows: 0, 1, and 2: negative; and 3, 4, and 5: positive.

### Statistical analysis

The effects of JTE-522 on pulmonary metastasis were analyzed by the Kruskal-Wallis test and are expressed as means ± standard deviation. A correlation between COX-2 and VEGF expression was analyzed by Spearman's rank test. In all instances, statistical significance was established at p < 0.05.

## Results

### Pulmonary metastases

**Figure 1:**
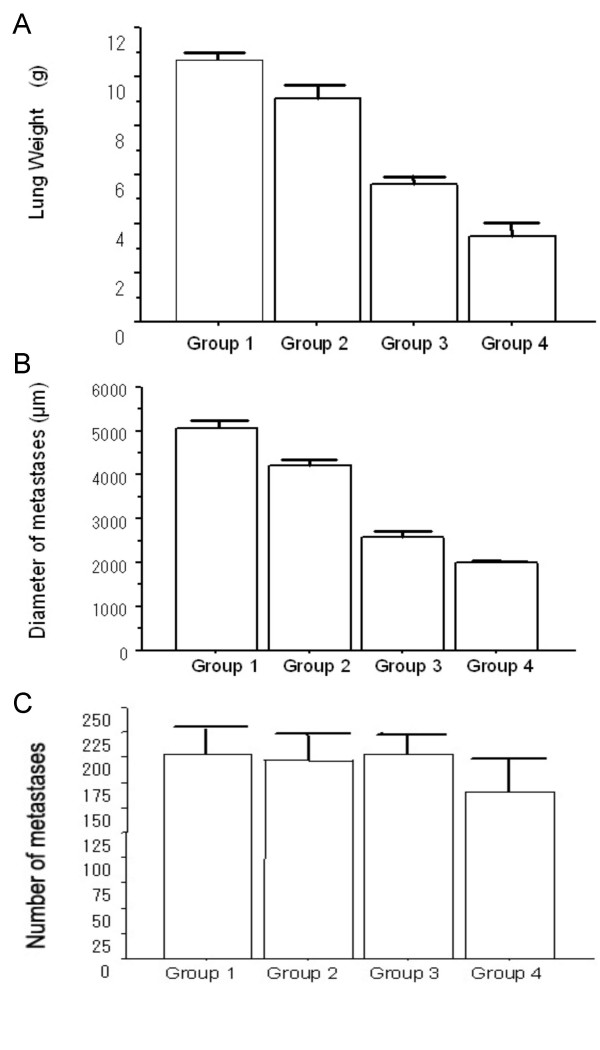
**Graphs of pulmonary metastases of rats. **(A) Lung weight of control and JTE-522 treated rats injected with RCN-9 (group 1, 10.70 ± 0.57 g; group 2, 9.14 ± 0.62 g; group 3, 5.61 ± 0.76 g; group 4, 3.52 ± 0.81 g). JTE-522 significantly and dose-dependently decreased the lung weight of each group (p = 0.0001). (B) Maximal diameter of metastatic tumors in control and JTE-522 treated rats injected with RCN-9 (group 1, 5067 ± 499 μm; group 2, 4233 ± 244 μm; group 3, 2583 ± 211 μm; group 4; 1990 ± 96 μm). JTE-522 significantly and dose-dependently decreased size of metastases (p = 0.0002). (C) The number of metastatic tumors in control and JTE-522 treated rats injected with RCN-9 (group 1, 203 ± 26; group 2, 197 ± 26; group 3, 204 ± 18; group 4, 165 ± 33). Microscopy shows no significant differences in the number of metastases (p = 0.13)

**Figure 2:**
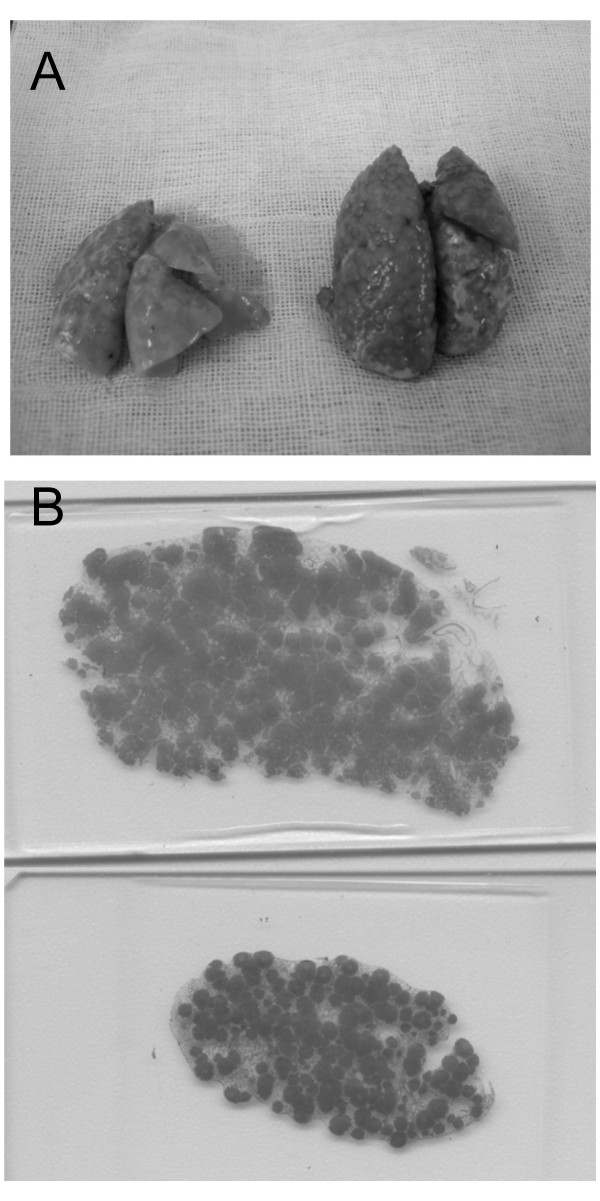
**Removed lungs and the largest cross sections. **(A) Representative lungs of rats injected with RCN-9. Removed lungs on the left were those of group 4 (JTE-522: 30 mg/kg/day); and those on the right were group 1 (control). Surface of the lungs of group 1 has more metastatic tumors than that of group 4. (B) The largest cross-sections of group 1 (control) and group 4 (JTE-522: 30 mg/kg/day), which were stained with hematoxylin and eosin. The upper slide shows the largest cross-section of group 1, and the lower one shows that of group 4. Many metastatic tumors are not visible on the surface.

Each group consisted of 6 rats. JTE-522 significantly and dose-dependently decreased the weight of the lungs (p = 0.0001, Figure 1A). Cross sections contained many metastatic tumors that were not visible on the surface (Figures 2). The size of the metastatic tumors was significantly and dose-dependently decreased (p = 0.0002, Figure 1B), although the number of metastases did not differ (Figure 1C). JTE-522 appeared to have no significant adverse effects.

### Immunohistochemistry

**Figure 3:**
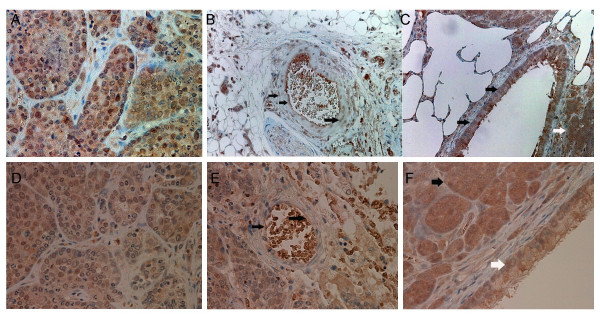
**Immunohistochemical staining for COX-2 and VEGF. **A, B, and C show immunohistochemical staining for COX-2 in lungs of rats injected with RCN-9. A, group 1 (control group); B and C, group 4 (JTE-522; 30 mg/kg/day). Tumor cells in all groups expressed COX-2. Degree of staining between groups 1 and 4 did not differ. Vascular structures (black arrow, B) and large airways (black arrow, C) in the rat lung were stained for COX-2. White arrow in C shows tumor cells stained for COX-2. Original magnification, A and B, ×400, C, ×200. D, E, and F show immunohistochemical staining for VEGF in lungs of rats injected with RCN-9. D and E, group 1 (control); F, group 2 (JTE-522; 3 mg/kg/day). Tumor cells of all groups expressed VEGF. Degrees of staining between groups 1 and 2 did not differ (D and F). Vascular structures (black arrow, E) and large airways (white arrow, F) were stained for VEGF in rat lung. Black arrow in F shows tumor cells stained for VEGF. Original magnification, ×400.

**Figure 4:**
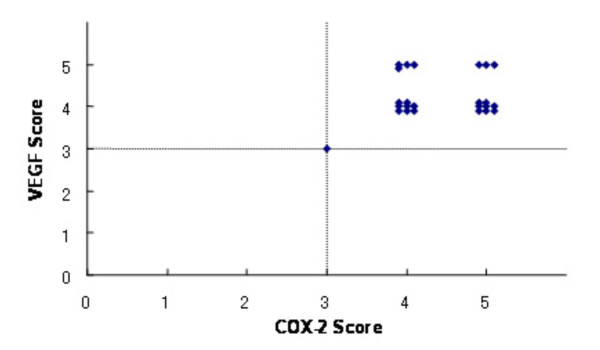
**Immunohistochemical score of COX-2 and VEGF. **Relation between the immunohistochemical score of COX-2 and that of VEGF is shown. The score was defined as positive, if it was 3 or more. The number of each score (COX-2 vs VEGF) was as follows: 3 vs 3, 1; 4 vs 4, 8; 4 vs 5, 4; 5 vs 4, 8; and 5 vs 5, 3. Both COX-2 and VEGF were positive in all groups, however, there was no correlation between COX-2 and VEGF scores (p = 0.60, Spearman's rank test).

Immunohistochemical staining showed that both COX-2 and VEGF were expressed at high levels in pulmonary metastatic tumors of all groups (Figures 3). The large airways and vascular structures were stained for both proteins, as were tumor cells in all groups. However, there was no correlation between COX-2 score and VEGF score (p = 0.60, Figure 4). The degree of COX-2 and VEGF immunohistochemical staining in tumor cells did not significantly differ among groups (Table 1).

## Discussion

JTE-522 dose-dependently reduced the weight of the lung with metastases and the size of metastatic tumors. These observations are consistent with those of other studies of selective COX-2 inhibitors. In the present study, however, the number of metastatic lesions determined microscopically was not reduced by JTE-522, which conflicts with the earlier findings. Masferrer *et al*. described the inhibition of tumor growth and lung metastasis of Lewis Lung carcinoma in mice by continuous dietary celecoxib (160–3200 ppm) supplied from the date of implantation [18]. Celecoxib at a dose of 160 ppm did not affect the number and size of lung metastases, but doses between 480 and 3200 ppm reduced the number of metastatic nodules by >50%. Histopathological analysis revealed that celecoxib dose-dependently reduced the size of the metastatic lung tumors. Masferrer *et al*. evaluated the pharmacological effects of celecoxib on lung metastasis by counting the metastatic tumors using a stereomicroscope and by histochemical analyses of consecutive lung sections. Tomozawa *et al*. investigated the effect of JTE-522 on lung metastasis of colon cancer in mice [19]. Their study showed that JTE-522 significantly reduced the number of metastatic nodules and lung weight. They macroscopically counted the metastatic tumors on the lung surface. Our study using cross sections disclosed that many nodules were not visible on the surface and could not be seen by the naked eye. These observations may indicate that JTE-522 blocks the growth of metastatic tumors, but does not inhibit the process of metastasis. On the other hand, a recent study in vitro, using cancer cell lines preincubated with celecoxib, suggests that COX-2 is involved in adhesion of cancer cells to vascular endothelium [20]. If the cancer cells had been pretreated with JTE-522 for an appropriate period in the present study, it might reduce the number of the lung metastases.

Here, we immunohistochemically stained COX-2 and VEGF because some reports indicate that prostaglandins regulate VEGF expression [21,22] and that COX-2 inhibitors directly affect angiogenesis [23]. Other recent studies have confirmed the notion that tumor growth and metastasis are dependent on angiogenesis and require the development of new vessels [24-26], and that COX-2 is related to tumor angiogenesis. COX-2 modulates the production of angiogenic factors derived from colon cancer cells, while COX-1 regulates angiogenesis in endothelial cells [27]. COX-2 is also related to tumor angiogenesis in human colorectal cancer. Cianchi *et al*. identified a significant correlation between COX-2 and VEGF, the latter of which might be one of the most important mediators of the COX-2 angiogenic pathway [28]. Immunohistochemical staining in the present study revealed parallel COX-2 and VEGF expression that was mainly localized in the cytoplasm of tumor cells, epithelial cells of the airways, and vascular structures. These findings may indicate that COX-2 is involved in the production of VEGF. Furthermore, the present study supports our previous report that JTE-522 interferes with the growth of metastatic tumors of colon cancer, due to inhibition of neovascularization [29]. Our results showed that high levels of COX-2 were expressed in the groups administered with JTE-522 as well as in controls. This is consistent with the fact that JTE-522 suppresses not the production, but the activity of COX-2 in prostaglandin biosynthesis [15]. VEGF was also highly expressed in all groups. One of the reasons may be hypoxia induced by multiple pulmonary metastases. Christou *et al*. reported that hypoxic animals have significantly higher VEGF concentrations compared with normoxic controls [30]. On the other hand, these findings may indicate that VEGF is produced via a pathway, which does not include COX-2, as well as through a COX-2 pathway.

We reported that COX-2 is related to human colorectal tumorigenesis. COX-2 mRNA expression is obviously increased in sporadic adenomas compared with normal colorectal mucosa and is significantly greater in adenomas with larger diameters [31]. Additionally, COX-2 expression and degree of dysplasia are significantly associated in adenomas [32]. COX-2 levels in human colorectal carcinomas are significantly higher in larger tumors and in those that invade more deeply [12]. We therefore postulated that COX-2 might correlate with metastasis as well as primary colorectal tumor, because hematogenous metastasis is the key for prognostic factor in colorectal cancer. The advances in the treatment for metastasis would improve the prognosis of the patients with colorectal cancer. The present study found that JTE-522 dose-dependently suppressed metastatic tumor growth, although the function to prevent cancer cells from metastasizing might not be so strong. Our recent report has revealed that administration of rofecoxib, a selective COX-2 inhibitor, decreased the number and size of rectal polyps in familial adenomatous polyposis patients, whereas the relapse of rectal polyps was seen 3 months after completion of the treatment [33]. This phenomenon might indicate that COX-2 inhibitors make colorectal polyps invisible, although atypical cells still exist microscopically. This is consistent with the result of the present study.

Recent reports have shown the inhibitory effect of rofecoxib on liver metastases. Yao *et al*. demonstrated that rofecoxib decreased the growth and metastatic potential of colorectal cancer in mice through multiple mechanisms [34]. Fenwick *et al*. reported that refecoxib negatively regulated angiogenesis in human colorectal cancer liver metastases [35]. It is considered that COX-2 inhibitors can be used as therapeutic agents for hematogenous metastases of colorectal cancer.

## Conclusion

In conclusion, JTE-522, a selective COX-2 inhibitor, dose-dependently inhibited the growth of pulmonary metastasis from colorectal cancer in a rat model. Selective COX-2 inhibitors may constitute useful therapeutic agents that can interfere with the growth of hematogenous metastasis, as well as the tumorigenesis of colorectal cancer.

## Competing interests

The author(s) declare that they have no competing interests.

## Authors' contributions

HK carried out the animal experiment and drafted the manuscript. HK and TH performed immunostaining and its evaluation. HU performed the statistical analysis. ME and KS participated in the design of the study and its coordination. All authors read and approved the final manuscript.

## Pre-publication history

The pre-publication history for this paper can be accessed here:



## Figures and Tables

**Table 1:** Immunohistochemical staining score of COX-2 and VEGF.

Group	COX-2 Expression Score	VEGF Expression Score
Group 1	4.5 ± 0.55	4.0 ± 0.51
Group 2	4.5 ± 0.55	4.0 ± 0.41
Group 3	4.5 ± 0.55	4.0 ± 0.41
Group 4	4.0 ± 0.75	4.0 ± 0.82
p value	NS	NS

NS: not significant
